# Induction of ROS and DNA damage-dependent senescence by icaritin contributes to its antitumor activity in hepatocellular carcinoma cells

**DOI:** 10.1080/13880209.2019.1628073

**Published:** 2019-08-13

**Authors:** Shikang Wang, Qian Wang, Huijun Wang, Chengkun Qin, Xianping Cui, Lei Li, Yongqing Liu, Hong Chang

**Affiliations:** a Emergency Center, Shandong Provincial Hospital Affiliated to Shandong University, Jinan, China;; b Department of Hepatobiliary Surgery, Shandong Provincial Hospital Affiliated to Shandong University, Jinan, China;; c Institute of Medical Sciences, The Second Hospital of Shandong University, Jinan, China;; d Department of Internal Medicine, Shandong Cancer Hospital Affiliated to Shandong University, Shandong Academy of Medical Sciences, Jinan, China;; e Department of Clinical Pharmacy, The Second Hospital of Shandong University, Jinan, China

**Keywords:** Cellular senescence, senescence-associated-β-galactosidase activity, ROS-induced DNA damage, prenylflavonoid derivative

## Abstract

**Context:** Icaritin (ICT), a prenylflavonoid derivative extracted from the *Epimedium* (Berberidaceae) genus, has been identified to exhibit antitumor effect in hepatocellular carcinoma (HCC) cells by inducing apoptosis. However, its effect on cellular senescence has not been elucidated.

**Objective:** To investigate the mechanism for low concentrations of ICT exerting antitumor activity through induction of cellular senescence.

**Materials and methods:** Human HepG2 and Huh7 cells were treated with low concentrations of ICT (1 and 2 μM) once per day for a week. Cellular senescence was evaluated through cell viability and senescence-associated-β-galactosidase activity. Cell cycle distribution and ROS levels were measured with flow cytometry. Gene expression was detected using qRT-PCR and western blotting. Fluorescent punctuates formation of γH2AX was analyzed by immunofluorescence.

**Results:** ICT (1 and 2 μM) promoted cellular senescence in HepG2 and Huh7 cells, as observed by enlarged and flattened morphology and increased senescence-associated-β-galactosidase activity (∼7-8-fold and ∼11-12-fold of vehicle controls, respectively), accompanied by significant cell cycle arrest and decrease in DNA synthesis. Mechanistically, ICT-induced senescence occurred through accumulation of ROS (∼1.3-fold and ∼1.8-fold of vehicle controls in response to 1 and 2 μM ICT, respectively), which further resulted in DNA damage response, as evidenced by strong induction of γH2AX through immunofluorescence and western blotting assays. Pharmacological inhibition of ROS production with *N*-acetylcysteine attenuated ICT-induced γH2AX and senescence-associated-β-galactosidase activity (∼0.28-0.30-fold decrease, *p* < 0.05).

**Discussion and conclusions:** Induction of cellular senescence by ICT defines a novel anticancer mechanism of ICT and provides a rationale for generalizing the study design to a broader study population to further developing ICT as a novel therapeutic agent for treatment of HCC.

## Introduction

Liver cancer is the sixth most common cancer and the second leading cause of cancer death worldwide (Torre et al. 2015). Hepatocellular carcinoma (HCC), accounting for 70–90% of primary liver cancers, has a poor prognosis with 5-year overall survival rates of <12% (Torre et al. 2015). Currently, surgical resection and transplantation remain the main approaches to treat HCC. However, most HCCs are inoperable due to advanced stages when surgery is no longer applicable, particularly in China and other Eastern countries (Torre et al. 2015; Chen et al. 2016). Further, after surgical resection, the long-term prognosis of HCC is still poor and development of cancer recurrence or metastasis remains a major problem (El-Serag and Rudolph 2007). Advanced stage HCC always shows weak response to traditional chemotherapeutic drugs, such as cisplatin and its relatives (Singh et al. 2014). In addition, acquired chemo resistance and drug toxicity in patients prevent long-term usage of these chemotherapeutic drugs (Singh et al. 2014). Thus, there is an urgent need to explore alternative approaches using effective and less toxic anti-HCC agents.

Cancer cells are driven to face their death and choose their subsequent fate following chemotherapy (Gonzale et al. 2016). These cell fate determinations include classical types of cell death, apoptosis and necroptosis, and therapy-induced cellular senescence. Senescence is defined as a state in which an irreversible permanent growth arrest occurs accompanied by changes in cell morphology and physiology. Senescent cells remain viable and metabolically active but losing proliferation potential and the ability for DNA synthesis and further cell division (Grimes and Chandra 2009; Campisi 2013), thereby distinguishing senescence from cell death. Senescent cells display characteristic changes, typically including cell cycle arrest and increased senescence-associated-β-galactosidase (SA-β-Gal) activity, along with distinctive changes in morphology to flat and enlarged cell shape, the senescence-associated secretory phenotype (SASP), and expression alterations in the molecular biomarkers (Campisi 2013). Senescence can be prematurely triggered by various inappropriate stresses which include telomere dysfunction, oncogene activation, DNA damage, ROS and chromatin perturbation (Rodier and Campisi 2011). Recently, accumulating data have demonstrated that senescence is a potent tumour suppressive mechanism to bar the initiation and development of cancer, and senescence-based chemotherapy, as a possible therapeutic option, has been receiving increasing attention (Cairney et al. 2012; Sagiv and Krizhanovsky 2013).

Icaritin (ICT) is a prenylflavonoid derivative from *Epimedium brevicornu* Maxim. (Berberidaceae), a traditional Chinese Herbal medicine. Previous studies have shown that ICT could exert a number of biological activities and health benefits, including induction of differentiation of various cells, protection of neuronal cells, and prevention against steroid-associated osteonecrosis (Zhu and Lou 2005; Wang et al. 2007; Yao et al. 2012). Recently, ICT’s activities in cancer chemoprevention were given increased attention. It was found that ICT exhibits antitumor effects in human cell lines established from breast cancer, prostate cancer, endometrial cancer, renal cell carcinoma, and leukaemia (Huang et al. 2007; Guo et al. 2011; Tong et al. 2011; Zhu et al. 2011; Li et al. 2013). The activity of ICT in HCC was also tested by previous studies, which mainly focussed on its cytotoxic effects by induction of apoptosis (He et al. 2010; Sun et al. 2015). Whether ICT exerts antitumor activity via cellular senescence has not been demonstrated. In the present study, we found that apoptosis was not the sole mechanism by which ICT inhibited tumour cell growth because low concentrations of ICT induced cellular senescence in HCC cells. Further investigations demonstrated that ICT induced production of ROS and DNA damage, which played a crucial role in ICT-mediated cellular senescence.

## Materials and methods

### Cell culture and treatments

The human HCC cell lines HepG2 and Huh7, and normal human liver cell line L02, were obtained from the Cell Bank of Chinese Academy of Sciences (Shanghai, China) and maintained in MEM, DMEM, and DMEM medium (HyClone, Thermo Fisher Scientific, USA) supplemented with 10% foetal bovine serum (HyClone), respectively. The cells were maintained in a humidified incubator with 5% CO_2_ at 37 °C.

ICT (Sigma-Aldrich, St. Louis, MO, USA) was prepared in dimethylsulphoxide (DMSO, Sigma-Aldrich) at 50 mM as a stock solution stored at −20 °C. Final desired concentrations (0.2–10 μM) were diluted with the medium before each experiment. In some experiments, cells were exposed to 2 mM of the antioxidant *N*-acetylcysteine (NAC; Sigma-Aldrich) for 1 h before treatment with ICT.

### MTT assay

MTT [3-(4,5-dimethylthiazol-2-yl)-2,5-diphenyl-2H-tetrazolium bromide, Sigma-Aldrich] assay was performed to quantitatively detect cell viability in the presence of ICT. HCC cells were seeded in 96-well culture plates and challenged with ICT (0.2, 0.5, 1, 2, 5, 10 μM) for 1 week. Then cells were incubated with 10 μL MTT for 4 h and the absorbance at 570 nm was determined on a plate reader (Bio-Rad, USA).

### Colony formation assay

The HCC cell lines HepG2 and Huh7 were seeded into 6-well plates (1000 cells/well) and treated with 1 and 2 μM ICT or vehicle. After fixed using methanol, the clones were stained with Giemsa. The number of clones was counted and the inhibition rates of colony formation were calculated compared to the vehicle control.

### 5-Bromo-2-deoxyuridin (BrdU) incorporation assay

DNA synthesis response of HCC cells to ICT was detected via BrdU incorporation assay by using the BrdU Cell Proliferation Kit (Millipore, DT, Germany). Cells were seeded into 96-well plate and incubated with 1 and 2 μM ICT for 1 week. Then the cells were treated with BrdU for an additional 8 h, and spectrophotometric detection was performed according to the manufacturer’s protocol.

### Cell cycle analysis

The HCC cell lines HepG2 and Huh7 were seeded in 6-well plates and exposed to 1 and 2 μM ICT or vehicle. Then the cells were collected, washed with ice-cold PBS, and then fixed in iced 70% ethanol. After incubated with RNase (Invitrogen, CA, USA) and propidium iodide (PI, Sigma-Aldrich), cell cycle analysis was performed by flow cytometry (Becton Dickinson, NJ, USA).

### Western blotting assay

After treatment with 1 and 2 μM ICT, whole cell lysates were prepared with RIPA buffer (Liu et al. 2019). Proteins were quantified using the BCA protein assay (Beyotime Institute of Biotechnology, Shanghai, China). Samples containing equal amounts of protein (60 μg) from the lysates were separated by SDS-PAGE and transferred to nitrocellulose membrane (GE Healthcare, CT, USA). After being blocked with 5% non-fat milk, blots were incubated with primary antibodies against glyceraldehyde 3-phosphate dehydrogenase (GAPDH), p21 (Santa Cruz Biotechnology, TX, USA), and phosphor-H2AX (Ser139, γH2AX) overnight at 4 °C, respectively, and then incubated with peroxidase-conjugated appropriate secondary antibodies. Membranes were stripped and immunoblot bands were visualized using an enhanced chemiluminescence (Millipore). GAPDH served as a protein loading control.

### Quantitative real-time PCR (qRT-PCR) analysis

Total RNA of HepG2 and Huh7 cells treated with ICT were extracted by an RNAiso plus kit (Takara Bio, Inc., Otsu, Japan) according to the manufacturer’s instructions. cDNA was synthesized using a PrimeScript RT reagent Kit (Takara Bio, Inc.). qRT-PCR was carried out using a Real-time PCR System (Eppendorf International, Germany). The sequences of the primers were as follows: IL-6, 5′-ACTCACCTCTTCAGAACGAATTG-3′ (forward) and 5′-CCATCTTTGGAAGGTTCAGGTTG-3′ (reverse); IL-8, 5′-CTGAGAGTGATTGAGAGTGGAC-3′ (forward) and 5′-ACCCTCTGCACCCAGTTTTC-3′ (reverse); GAPDH, 5′-TGGTCACCAGGGCTGCTT-3′ (forward) and 5′-AGCTTCCCGTTCTCAGCCTT-3′ (reverse), respectively. Changes in the mRNA levels of IL-6 and IL-8 were normalized to the level of GAPDH.

### Immunofluorescence assay for γH2AX

Cells were grown on cover-slips and treated with 1 and 2 μM ICT as described. Then the treated cells were fixed with methanol/acetone (1:1) followed by incubation with 3% BSA. Next, the cells were incubated with primary antibody against γH2AX and immunostained with the secondary antibody. Nuclei were stained with DAPI (Sigma-Aldrich). The fluorescence images were captured using a confocal microscope (Carl Zeiss, LSM780, Germany).

### ROS measurement

The production of ROS induced by 1 and 2 μM ICT was monitored using the fluorescent dye hydroethidine 2′,7′-dichlorodihydrofluorescein diacetate (H(2)DCFDA, Molecular Probes, Sigma-Aldrich). After treatment with ICT for the indicated times, cells were incubated with 10 μM H(2)DCFDA at 37 °C for 30 min. Then the cells were washed with PBS buffer and subjected for measurement of ROS using flow cytometry (Becton Dickinson, Franklin Lakes, NJ, USA).

### Statistical analysis

The data were presented as the mean ± SD. The statistical significance of the mean differences between the control and treated groups was determined with 2-tailed Student’s *t*-test. Multiple group comparisons were performed with one-way ANOVAs, followed by Dunnett’s multiple comparison test. For all tests, *p* < 0.05 was considered to be statistically significant.

## Results

### Low concentrations of ICT suppress HCC cell proliferation and induce cellular senescence

**Figure 1.**
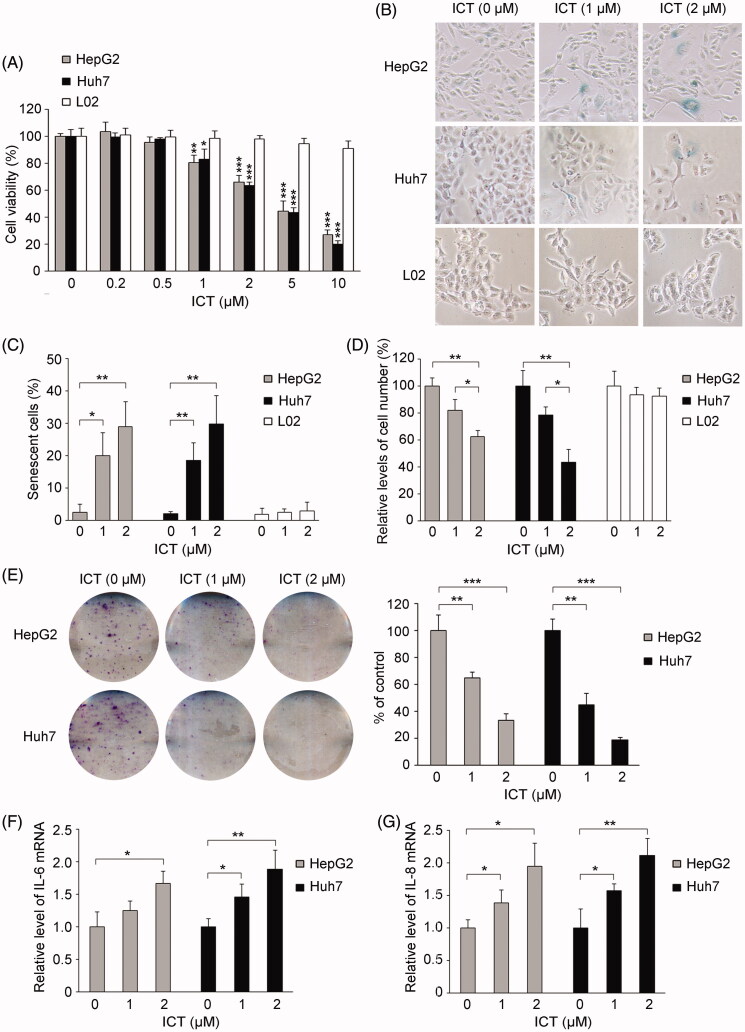
Low concentrations of ICT induce cellular senescence in human HCC cells. (A) HepG2, Huh7 and L02 cells were treated with the indicated doses of ICT, and cell viability was analyzed by MTT assay. (B-D) SA-β-Gal staining in ICT-treated HepG2, Huh7 and L02 cells. Senescent cells were observed (B), and the percentages of SA-β-Gal-positive cells (C) as well as the inhibitory effect of ICT on the growth of cell number (D) were shown. (E) ICT inhibited colony formation of HepG2 and Huh7 cells. The representative images and quantitative analysis of foci formation were shown. (F and G) qRT-PCR analysis of IL-6 and IL-8 expression in HepG2 and Huh7 cells after ICT treatment. In (A, C-G), results are the mean ± SD of three independent experiments. *p < 0.05, **p < 0.01 and ***p < 0.001 versus ICT-untreated control group, respectively.

To explore whether ICT induced premature senescence and determine its dosages to induce senescence in HCC cells, we first performed cell viability and SA-β-Gal analysis. It has been demonstrated that ICT at 5–40 μM concentrations, is sufficient to induce apoptosis in HCC cells (He et al. 2010; Sun et al. 2015). Interestingly, we found here ICT also effectively inhibited cell proliferation at low concentrations about 1–2 μM after long-time treatment in both human HCC cells HepG2 and Huh7 (Figure 1(A)). In contrast, the result showed that L02 normal human hepatocytes, as controls, were more resistant to low concentrations of ICT capable of inhibiting cell viability (Figure 1(A), *p* > 0.05). Meanwhile, as shown in Figure 1(B–D), the result of SA-β-Gal staining showed that low concentrations of ICT at 1-2 µM indeed induced cellular senescence in HepG2 and Huh7 cells as evidenced by increased SA-β-Gal positive cells, companied with clear accumulation of cells with enlarged and flattened morphology. Moreover, the concentration-dependent senescence-inducing effect of ICT was observed, with significant enhancement of the SA-β-Gal positive stained cells by ∼7–8-fold and ∼11–12-fold in both cells treated with 1 and 2 μM ICT, respectively. Meanwhile, in corresponding to the results in Figure 1(A), the inhibitory effect of ICT on HCC cells was also evidenced by the decrease in cell number after a week treatment as shown in Figure 1(B,D). Whereas, 1–2 μM ICT did not display significant senescence-inducing effect on L02 cells, which was in keeping with the observation in Figure 1(A) that L02 cells presented a decrease in sensitivity to ICT when compared to HCC cells. Further, colony formation assay also showed that treatment with ICT significantly inhibited the colony-forming potential in a concentration-dependent manner, and the foci number decreased ∼40–50% and ∼70–80% in response to 1 and 2 μM ICT in both cells respectively, significantly lower than that of vehicle-treated controls (Figure 1(E), *p* < 0.01 and *p* < 0.001 respectively). Additionally, the senescence-associated secretory phenotype (SASP) was measured. As shown in Figure 1(F,G), expression levels of SASP markers IL-6 and IL-8 were found significantly increased in both HCC cells after ICT treatment. Together, the data demonstrated that low concentrations of ICT triggered premature senescence in HCC cells.

### ICT induces cell cycle arrest, inhibits DNA synthesis and modulates senescence-associated proteins

**Figure 2.**
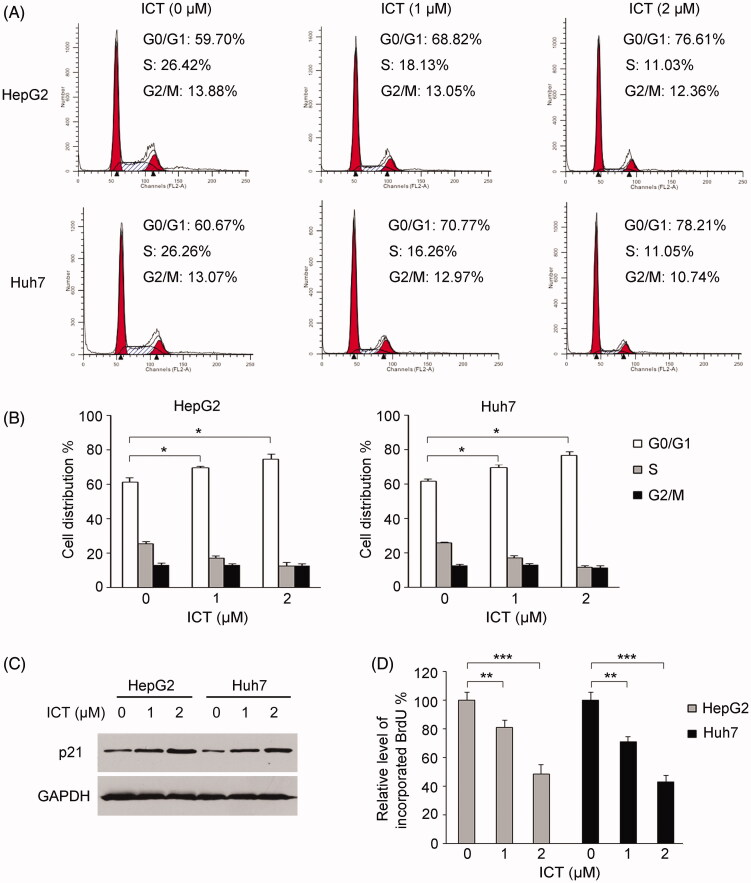
Effect of ICT on cell cycle and DNA synthesis. (A and B) Cell cycle arrest induced by ICT was analyzed by flow cytometry and quantitative analyses of per cent gated cells at the G0/G1, S and G2/M phases were shown. (C) Western blotting analysis of p21 in ICT-treated HepG2 and Huh7 cells. (D) Effect of ICT on BrdU incorporation into cells. The percentages of cells that incorporated BrdU into DNA were shown. In (B and D), results are presented as the mean ± SD of three independent experiments. *p < 0.05, **p < 0.01 and ***p < 0.001 compared to ICT-untreated control group, respectively.

As cell cycle arrest and DNA synthesis inhibition were also characteristic changes during cellular senescence, we then assessed the impact of ICT on cell cycle and DNA synthesis to validate ICT-induced senescence. The result of flow cytometry revealed that, compared with vehicle controls, treatment with low concentrations of ICT led to a significant accumulation of cells in G0/G1 phase, with a corresponding decrease in S and G2/M-phase fractions (Figure 2(A,B). The percentages of HepG2 cells in G0/G1 phase were observed to be 59.70% in control-treated HepG2 cells, while 1 and 2 μM ICT treatment increased the percentages to 68.82% and 76.61% (*p* < 0.05), respectively. Similarly, 70.77% and 78.21% of Huh7 cells in the G0/G1 phase were enforced following 1 and 2 μM ICT treatment in comparison with 60.67% in control cells (*p* < 0.05). Concordantly, ICT significantly enhanced the expression of the cyclin-dependent kinase inhibitor p21 in HepG2 and Huh7 cells (Figure 2(C)). Since p21 is critical for controlling G1/S transition and cellular senescence (Zhang 2007; Deng et al. 2008), alteration in p21 was favouring cell cycle arrest at G0/G1 phase, which was corresponding to the result observed in the flow cytometry analysis. Moreover, BrdU incorporation assay was performed to analyze the effect of ICT on DNA synthesis. The result showed that ICT treatment noticeably reduced the BrdU incorporation in a concentration-dependent manner (Figure 2(D)). Treatment with 1 and 2 μM ICT downregulated the BrdU incorporation in HepG2 and Huh7 cells to ∼56.8–58.1% and ∼33.9–35.1% compared with vehicle control cells, respectively. Thus, ICT-induced cell cycle arrest and DNA synthesis reduction further confirmed its ability to trigger senescence.

### ICT induces ROS generation and subsequent activation of DNA damage

**Figure 3.**
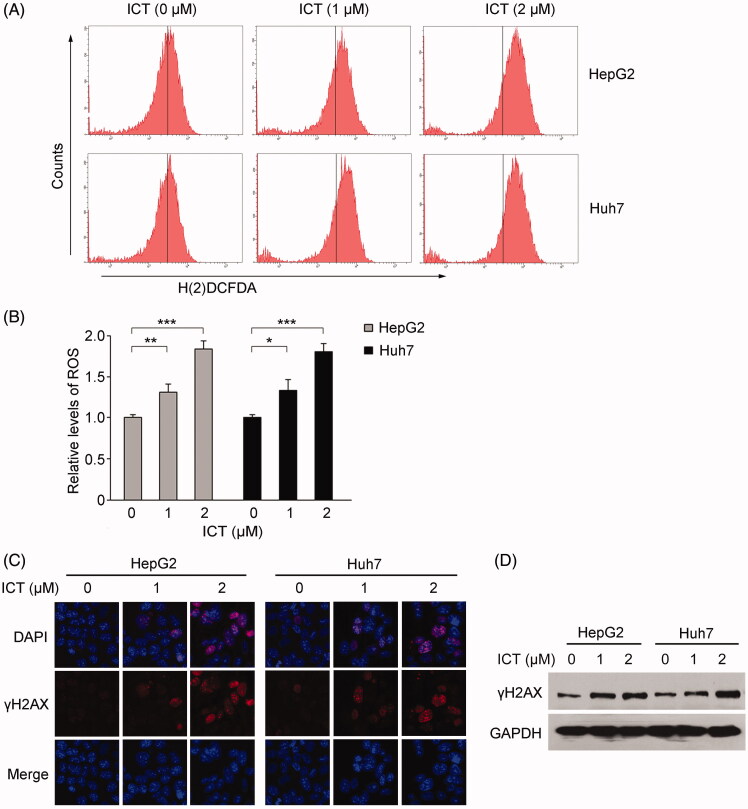
Effect of ICT on the generation of ROS and DNA damage. (A) Effect of ICT on the generation of ROS. The ROS levels in ICT-treated HepG2 and Huh7 cells were detected by H(2)DCFDA staining and flow cytometry. (B) Statistical analysis of the relative ratio of ROS formation. Results are the mean ± SD of three independent experiments. *p < 0.05, **p < 0.01 and ***p < 0.001 compared to ICT-untreated control group, respectively. (C) Immunofluorescence of γH2AX foci in response to ICT in HepG2 and Huh7 cells. Nuclei were counterstained with DAPI. (D) Western blotting analysis of γH2AX. GAPDH served as the loading control.

Since excessive ROS are widely accepted to stimulate cellular senescence (Correia-Melo et al. 2016; Kudryavtseva et al. 2016). Thus, we evaluated whether ICT interfered with ROS generation by using a fluorescent probe H(2)DCFDA. The intracellular accumulation of ROS induced by ICT was determined by calculating the geometric mean (Gmean) of fluorescence intensity in the affected cells with flow cytometry. As shown in Figure 3(A,B), ICT concentration-dependently increased ROS generation in HepG2 and Huh7 cells, with ∼30% increase for 1 μM ICT, and ∼80% increase for 2 μM ICT, respectively. The data indicated that low concentrations of ICT increased ROS level in HCC cells.

Because the generation of ROS leads to DNA damage and the accumulation of DNA damage is believed to be one of major drivers of premature senescence (Garinis et al. 2008; Campisi 2013), we also sought to examine the ability of ICT at lower dose to induce DNA damage. The foci assay of γH2AX, a well-known indicator in response to DNA damage (Zhang et al. 2016) was performed after treatment with ICT. In agreement with the changes in ROS production, it was observed in Figure 3(C) that evident fluorescent punctuate formation of γH2AX was accumulated in ICT-treated cells, while vehicle-treated controls presented negative fluorescent intensity. As shown in Figure 3(D), western blotting analysis also revealed ICT enhanced the expression level of γH2AX, in line with the results of immunofluorescence. The effect of ICT on the expression of γH2AX demonstrated a similar pattern in HepG2 and Huh7 cells, suggesting that ICT indeed induced DNA damage in HCC cells.

### Induction of DNA damage by elevated ROS level contributes to ICT-mediated senescence

**Figure 4.**
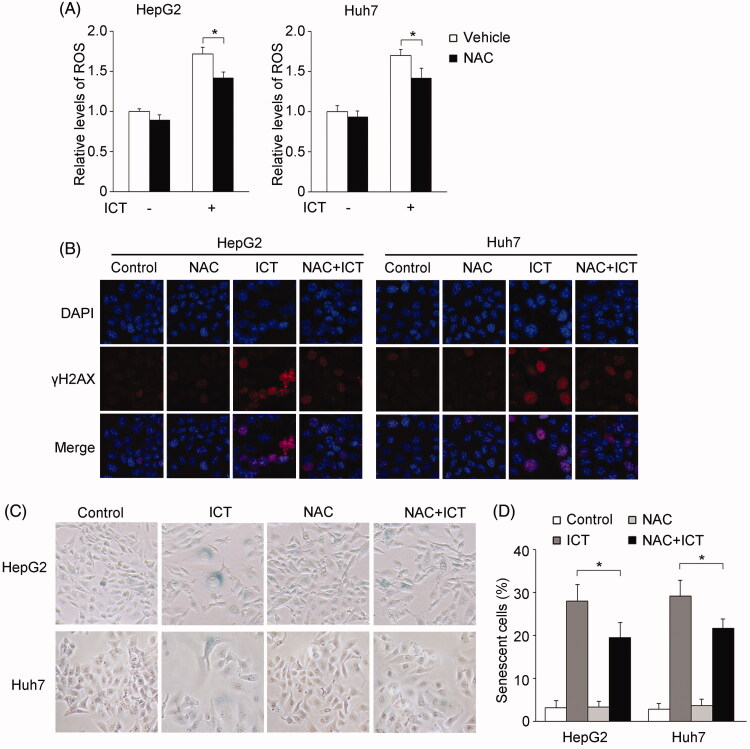
ICT-induced ROS and DNA damage trigger cellular senescence. (A) Effect of the antioxidant NAC on ICT-induced ROS generation. HepG2 and Huh7 cells were pre-treated with 2 mM NAC or vehicle prior to treatment with ICT. ROS production was then assessed by flow cytometry. (B) HepG2 and Huh7 cells were pre-treated with NAC or vehicle and subsequently incubated with ICT in the presence or absence of NAC. Then immunofluorescence staining of γH2AX foci was performed. Nuclei were counterstained with DAPI. (C and D) The senescent cells were detected by SA-β-Gal analysis after NAC and ICT treatment in HepG2 and Huh7 cells. In (A and D), results are presented as the mean ± SD of three independent experiments. *p < 0.05 versus the NAC-untreated control group.

Additionally, we determined whether ROS production is involved in ICT-induced DNA damage and cellular senescence. We firstly tested the scavenging effect of the antioxidant NAC on ICT-induced ROS generation. The results showed that pre-incubated cells with NAC prior to ICT treatment significantly reduced the amount of ROS in HepG2 and Huh7 cells (Figure 4(A)). Then the effect of ICT-provoked ROS on DNA damage was analyzed. As shown in Figure 4(B), cells treated with ICT displayed predominant induction of γH2AX foci compared to the control, while the formation of γH2AX foci was noticeably abolished in both cells pre-treated with NAC in the presence of ICT, suggesting that ICT-induced DNA damage was a downstream event of high levels of ROS. We further tested senescent cells using SA-β-Gal staining in the presence of antioxidant NAC before exposure of ICT. NAC significantly attenuated ICT-triggered senescent cells as indicated by decrease in SA-β-Gal positive cells (Figure 4(C)). As summarized in (Figure 4(D)), SA-β-Gal staining decreased ∼0.30-fold in HepG2 (*p* < 0.05), and ∼0.28-fold in Huh7 cells (*p* < 0.05), respectively. Thus, these results revealed that ICT induced DNA damage by causing ROS generation, which in turn potentiated its activity of promoting cellular senescence.

## Discussion

ICT, a naturally occurring prenylflavonoid derivative from the *Epimedium* genus, has gained a lot of attention due to its potential antitumor effect through inducing apoptosis. The main finding of the present study is that low concentrations of ICT were identified for the first time to trigger cellular senescence instead of apoptosis. As demonstrated in this study, after a prolonged treatment with ICT, cells lost proliferation potential and exhibited significant cellular senescence phenotype as observed by cell cycle arrest, reduced DNA synthesis and increased SA-β-Gal activity. Furthermore, our results revealed that ICT triggered production of ROS and DNA damage, which were crucial for ICT-induced cellular senescence.

Recently, cellular senescence is recognized as a potent tumour-suppressor mechanism by arresting mitosis (Grimes and Chandra 2009; Cairney et al. 2012). Accumulating reports have highlighted cellular senescence as a determinant of the outcome of conventional anticancer therapies, and indicated that pro-senescence by chemotherapeutic compounds could be an alternative strategy for cancer treatment (Ewald et al. 2010; Lee and Lee 2014). In comparison with induction of cell death, total doses of therapeutic drugs can be markedly reduced to trigger cellular senescence, thereby providing a possibility to avoid the severe side effects resulted from injury to normal tissues while effectively evading oncogene initiation and limiting cancer cell proliferation (Ewald et al. 2010; Sagiv and Krizhanovsky 2013). A previous study demonstrated that ICT induced apoptosis in HCC cells via a caspase-dependent pathway (He et al. 2010; Sun et al. 2015). However, the contribution of senescence induction by ICT to antitumor effect has not been well elucidated. Here, we further found that in addition to apoptosis induction, ICT could also trigger cellular senescence in HCC cell lines. This antitumor mechanism is predominant when low concentrations of ICT were used. Thus, according to previous study and our present results, cancer cells displayed distinct modes of action in response to varied dosage of ICT treatment that is, inducing cellular senescence at low concentrations, or leading to apoptotic cell death at high concentrations. Indeed, many other naturally occurring phytochemicals, such as well-studied docetaxel, camptothecin and resveratrol, have been reported to have this drug concentration dependent switch between apoptosis and senescence (Han et al. 2002; Karpinich et al. 2002; Ewald et al. 2010; Lee and Lee 2014).

It is now accepted that elevated levels of ROS can lead to a premature senescence *in vitro* and *in vivo* regardless of the number of replications (Grimes and Chandra 2009). ROS are the by-products of cellular metabolism and are primarily generated in the mitochondria. During oxidative stress, the level of ROS is elevated. Increasing ROS levels, in turn, may damage mitochondria and also lead to accumulation of oxidative damage to other cellular components. In the present study, following a prolonged treatment with ICT at low concentrations, HCC cells presented significant accumulation of ROS. It is interesting to note that ICT possesses both pro-oxidant and anti-oxidant properties depending on the cell types, dosage and other experimental conditions (Liu et al. 2014; Wu et al. 2014; Li et al. 2016). Then our results demonstrated that ICT-induced ROS were involved in cellular senescence in HCC cells. The senescent cells generated by ICT decreased significantly when ROS were scavenged by using the antioxidant NAC, further suggesting that ICT-induced cellular senescence was, at least in part, in a ROS-dependent manner. Noticeably, it has been recently demonstrated that ROS induced by ICT promoted apoptosis in human cervical cancer cells (Chen et al. 2019). Compared with our experiment conditions, much higher concentrations of ICT (12.5–34 μM) were used in their study, increasing ∼10–20-fold. Meanwhile, cells were treated with ICT for shorter time (24-48 h). Due to the different treatment methods, it was reasonable that high concentrations of ICT triggered apoptosis instead of senescence. Thus, their results were not contradictory to ours, but further demonstrated the drug concentration-dependent switch between apoptosis and senescence as clarified above.

ROS also act as stressors of DNA damage, a major trigger of cellular senescence (Grimes and Chandra 2009; Campisi 2013). ROS cause severe genomic DNA damage by interacting extensively with nuclear DNA and are believed to be responsible for DNA double-strand lesions (Grimes and Chandra 2009; Correia-Melo et al. 2016; Kudryavtseva et al. 2016). Upon DNA damage response, a major cellular defence against cytotoxic DNA damage is initiated to drive cells to undergo apoptosis or senescence, depending on the extent of DNA lesions and cellular context. Of note, induction of p53, a major effector of DNA damage response, and its target p21, can result in irreversible arrest of cell cycle progression and the state of cellular senescence (Zhang 2007; Deng et al. 2008). Our results showed that ICT increased fluorescent punctuate formation of γH2AX and activated p53/p21 signalling, while NAC attenuated γH2AX expression after co-treatment of NAC and ICT. These findings implied that overproduction of ROS by ICT mediated DNA damage response, leading to the contribution to ICT-induced senescence in HCC cells. Moreover, since ROS are known as drivers of signalling networks important for the maintenance of the senescent phenotype (Correia-Melo et al. 2016), it is still necessary to further clarify the critical downstream mediators of the ROS signalling pathway in response to ICT.

In summary, these data suggest a novel mechanistic basis for the antitumor effect of ICT through induction of cellular senescence in HCC cells. Low concentrations of ICT induced ROS generation and subsequently stimulated ROS-mediated DNA damage response. These events strongly stimulated cellular senescence upon ICT exposure. Thus, this finding provides a rationale for development of ICT as a novel therapeutic agent for effective and safe treatment of HCC.
